# Supplementary material for: The causal relationship between the human gut microbiota and pyogenic arthritis: a Mendelian randomization study
Source: Front Cell Infect Microbiol. 2024 Nov 26;14:1452480. doi: 10.3389/fcimb.2024.1452480 (PMC11629706; doi:10.3389/fcimb.2024.1452480)
Supplement: Supplementary file 1 [file DataSheet1.zip › mendelian test/ebi-a-GCST90027620.csv_leave_one_out.pdf]

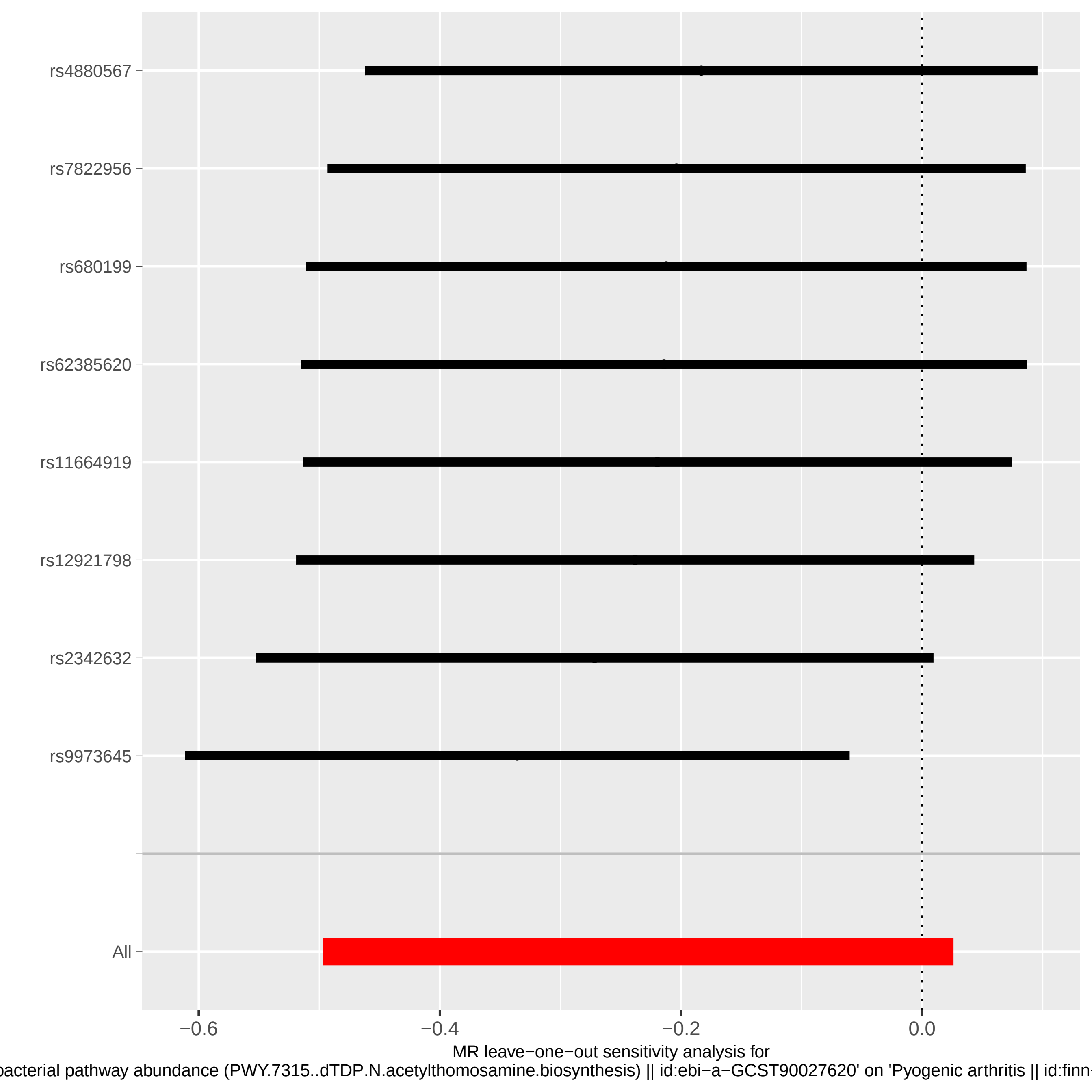
rs4880567
rs7822956
rs680199
rs62385620
rs11664919
rs12921798
rs2342632
rs9973645
All
−0.6
−0.4
−0.2
0.0
MR leave−one−out sensitivity analysis for
bacterial pathway abundance (PWY.7315..dTDP.N.acetylthomosamine.biosynthesis) || id:ebi−a−GCST90027620' on 'Pyogenic arthritis || id:finn-
